# Supplementary material for: Safeguarding children and strengthening families through the application and utilization of family group decision making: A systematic review protocol
Source: PLoS One. 2026 Apr 29;21(4):e0346794. doi: 10.1371/journal.pone.0346794 (PMC13127950; doi:10.1371/journal.pone.0346794)
Supplement: Attachment — Submitted filename: Rebuttal Letter for Resubmission to PLOS ONE.docx [file pone.0346794.s002.docx]

Rebuttal Letter for Resubmission to PLOS ONE

Date: September 27, 2025

To: The Editorial Team, PLOS ONE

Subject: Resubmission of Manuscript: Safeguarding Children and Strengthening Families through the Application and Utilization of Family Group Decision Making: A Systematic Review Protocol.

Dear Editorial Team,

Thank you for your review of our manuscript and for your feedback in your decision letter. We appreciate the opportunity to revise and resubmit our manuscript to PLOS ONE. We have addressed the concerns regarding the language restriction in our literature search, ensuring that our revised protocol aligns with PLOS ONE’s standards for systematic reviews.

We acknowledge your concern regarding our original submission's restriction of the literature search to English-language studies, which could introduce bias and affect the review's validity. We have revised the Eligibility Criteria section to provide a scientific rationale for this restriction. Our preliminary scoping search in EBSCOhost, PubMed, and Scopus revealed that approximately 85% of FGDM-related studies published between 2014 and 2024 are in English, with non-English studies largely consisting of case reports or regional studies that do not contribute unique FGDM methodologies or outcomes relevant to our objectives. Furthermore, FGDM originated in New Zealand and is predominantly adopted in English-speaking countries (e.g., UK, USA, Australia), where English-language publications are prevalent. To address potential bias, we have enhanced our grey literature search strategy to identify relevant non-English studies through expert consultation and reference checking, with documentation of these efforts in the final review. These updates are reflected in the revised Eligibility Criteria section (page 6) and discussed in the PRISMA checklist item 23c (page 13).

The revised protocol adopts a comprehensive and systematic approach to the literature review, as evidenced by our search strategy across multiple databases (Web of Science, EBSCOhost, PubMed, Sabinet, Scopus, Wiley Online Library) and the inclusion of grey literature. The methodology remains rigorous, with clearly articulated and reproducible methods for study identification, selection, and appraisal, utilizing Covidence, CASP checklists, and PRISMA guidelines. Additionally, we have addressed inconsistencies regarding the number of reviewers by aligning the protocol to specify three independent reviewers for screening, with a fourth reviewer designated to resolve conflicts, thereby enhancing transparency.

We believe that these revisions effectively address the concerns raised and enhance the protocol’s alignment with the guidelines established by PLOS ONE for systematic reviews. The revised manuscript and the updated PRISMA checklist have been submitted as a new submission, in accordance with your recommendations. Additionally, we have updated the Open Science Framework (OSF) registration DOI: <https://doi.org/10.17605/OSF.IO/B3TH8> to document these amendments.

We appreciate your consideration of our revised manuscript and look forward to the opportunity to contribute to PLOS ONE. Should you require any further clarification, please do not hesitate to contact me at [nmcollen@gmail.com](mailto:nmcollen@gmail.com) .

Sincerely,

Collen M. Ngadhi

University of the Western Cape

Centre for Interdisciplinary Studies of Children, Families, and Society

Cape Town, South Africa
