# Supplementary material for: Safeguarding children and strengthening families through the application and utilization of family group decision making: A systematic review protocol
Source: PLoS One. 2026 Apr 29;21(4):e0346794. doi: 10.1371/journal.pone.0346794 (PMC13127950; doi:10.1371/journal.pone.0346794)
Supplement: Attachment — Submitted filename: Responses to Reviewer.docx [file pone.0346794.s003.docx]

**To: The Editor**

**RE: Response to journal formatting requirements and reviewer comments**

**Manuscript title: Safeguarding children and strengthening families through the application and utilization of family group decision making: A systematic review protocol.**

Dear Editor,

We appreciate the opportunity to revise our manuscript titled “Safeguarding children and strengthening families through the application and utilization of family group decision making: A systematic review protocol.” We thank the reviewer for their insightful comments, which have substantially improved the rigor of our protocol. We have carefully addressed each suggestion and provide a detailed point-by-point response below.

**Journal requirements:**

1. The manuscript has been formatted in accordance with PLOSONE guidelines, including the title page structure (title, authors and affiliations denoted via superscript numerals, corresponding author contact information) and the overall layout as specified in the provided template.
2. We confirm the statement “The authors have declared that no competing interest exist.” This has been updated in the online submission form and included in the manuscript.
3. In accordance with protocol, no primary data were generated for this review. Extracted data from the final review will be made fully and openly available deposited in the Open Science Framework (OSF) repository linked to our protocol registration. This commitment is explicitly stated in the Data availability statement included in the manuscript (end of manuscript, before references).
4. While the reviewer did not suggest specific publications, we incorporated additional relevant references to provide greater specificity regarding developing countries. We thoroughly reviewed the reference list, ensuring all citations are complete and relevant. No retracted papers have been cited.
5. The reference list was reviewed for completeness and accuracy. No retracted publications were cited. Minor revisions including the addition of digital object identifiers (DOI) where available and the standardization of Vancouver-style formatting. The modifications are detailed in the tracked changes version of the manuscript.

**PLOS ONE Production Comment:**

We have amended the author information in the Editorial Manager submission form (via “Edit Submission”) to exactly match the manuscript title page.

We have added an in-text reference to Figure 1 in the Methods section (in data extraction) as requested: “The Preferred Reporting Items for Systematic Reviews and Meta-Analyses (PRISMA) flowchart will be employed to illustrate the identification, screening, eligibility, and inclusion phases of the systematic review process (See Figure 1 for details).

We have reviewed the file inventory and confirmed that only the most recent cover letter remains. All outdated versions have been removed.

**Reviewer comments:**

**Reviewer comment:** “There is a need to be specific concerning FGC focused on child care in developing countries.”

The authors express their gratitude to Prof. Shulamit Ramon for her positive assessment of the protocol’s rationale, technical soundness, feasibility, replicability, data availability and presentation. The suggestion to enhance specificity regarding Family Group Conference (FGC) focused on child care in developing countries is gratefully acknowledged. To address this limitation, the manuscript has been revised in certain sections to emphasize on application of FGDM/FGC in child safeguarding and family strengthening within developing country contexts. For instance, the abstract and introduction highlights unique prevalent in developing nations (resource limitations, informal support mechanisms and implementation deficits).

However, we wish to address an important methodological consideration regarding the search strategy. While the reviewer raises a valid point concerning the focus on developing countries, we must balance this specificity against the potential risk of excluding relevant literature that could inform developing country contexts. Limiting the scope to developing country terminology may inadvertently exclude studies conducted in developing nations that offer discussions of implications for low-resource setting or cultural adaptations pertinent to non-Western context. The methods section (eligibility criteria and search strategy) did not incorporate this level of specificity as it may limit the number of eligible studies, potentially resulting in a smaller sample size for inclusion.

To reconcile the reviewer's emphasis on developing country specificity with the need to avoid excluding potentially relevant evidence, a two-pronged methodological approach will be adopted. Firstly, a broad primary search without geographic restrictions will capture the entirety of the extant FGDM literature. This approach is essential because restricting the search to developing country terms risks excluding relevant studies from developed nations, such as research on cultural adaptations with minority populations, implementation in resource-constrained community settings, and insights into family engagement that transcend national boundaries. Secondly, a layered analysis framework will address the developing country focus. Initially, all studies from low- and middle-income countries (as classified by the World Bank) will be flagged for primary subgroup analysis, thus providing direct evidence from developing contexts. Subsequently, a secondary analysis will examine developed country studies to identify findings transferable to developing settings, including explicit implications for low-resource environments and cultural adaptations relevant to non-Western family structures. Lastly, a comparative analysis will identify similarities and differences in FGDM outcomes across contexts.

This revised approach is believed to comprehensively addresses the reviewer's concern whilst maintaining methodological rigor and maximizing the contribution of this review to the field.

We are confident that these revisions have strengthened the protocol and anticipate that the manuscript is now suitable for publication in *PLOS ONE*.

Thank you for your consideration.

Sincerely,

**Collen Mafira Ngadhi**

**On behalf of all authors**

**Corresponding author**

Email: [nmcollen@gmail.com](mailto:nmcollen@gmail.com)
